# Depression, anxiety symptoms, Insomnia, and coping during the COVID-19 pandemic period among individuals living with disabilities in Ethiopia, 2020

**DOI:** 10.1371/journal.pone.0244530

**Published:** 2020-12-30

**Authors:** Mogesie Necho, Mengesha Birkie, Habitam Gelaye, Abeba Beyene, Asmare Belete, Mekonnen Tsehay

**Affiliations:** Department of Psychiatry, Wollo University, College of Medicine and Health Sciences, Dessie, Ethiopia; Gachon University Gil Medical Center, REPUBLIC OF KOREA

## Abstract

**Background:**

People with disabilities face multiple barriers that prevent them from accessing care and essential information related to the COVID-19 pandemic that poses additional stress and psychopathology. Therefore, the investigation of psychopathologies during the COVID-19 outbreak and emergency response is critical.

**Methods:**

A cross-sectional survey was implemented from July 15/2020 to July 30/2020. The PHQ-9, GAD-7 scale, insomnia severity index-7, and brief resilient coping scale were administered to participants. The collected data was then entered into Epi-data version 3.1 and exported to SPSS-20 for analysis. Descriptive statistical procedures were employed to describe the various psychopathologies. A binary logistic regression method was used to identify the related factors for the psychopathologies. Furthermore, an odds ratio with its 95%CI was driven to show association strength, and a P-value <0.05 was declared as statistically significant.

**Results:**

A significant proportion of individuals living with disability had psychopathologies; 46.2% for depression symptoms, 48.1% for generalized anxiety disorder symptoms, and 71% for insomnia symptoms. Nearly 45.7% of participants were low resilient copers to their psychopathology. Depression was significantly higher in divorced/widowed/separated (AOR = 3.4, 95% CI: 1.28–8.92, P-value = 0.006), non-educated (AOR = 2.12, 95% CI: 1.12, 5.90, P-value = 0.001), and unemployed (AOR = 2.1, 95% CI: 1.32, 5.11, P-value = 0.005) as well as a daily laborer (AOR = 2.4, 95% CI: 1.20, 4.89, P-value = 0.014) subjects. Generalized anxiety disorder was also significantly higher in young age (<40 years) (AOR = 1.7, 95% CI: 1.32, 2.98, P-value = 0.02), single (AOR = 2.3, 95% CI: 1.24, 5.3, P-value = 0.011), widowed/divorced/separated (AOR = 1.5, 95% CI: 1.12, 2.78, P-value = 0.032), preparatory school completed (AOR = 3.00, 95% CI: 1.59, 5.46, P-value = 0.001), daily laborer (AOR = 2.7, 95% CI: 1.21, 5.23, P-value = 0.003), and unemployed (AOR = 2.5, 95% CI: 1.17, 4.78, P-value = 0.005) participants. Moreover, insomnia was significantly higher in single (AOR = 1.5, 95% CI: 1.12, 3.09, P-value = 0.027), divorced/widowed/separated(AOR = 6.2, 95% CI: 1.08, 11.29, P-value = 0.032), unemployed (AOR = 3.00, 95% CI: 1.22, 7.03, P-value = 0.001), blind (AOR = 2.8, 95% CI: 1.42, 6.35, P-value = 0.001), and deaf (AOR = 10.2, 95% CI: 4.52, 35.33, P-value = 0.002) participants.

**Conclusion:**

Depression, anxiety, and insomnia were highly prevalent among individuals with a disability during the COVID-19 period. Multiple sociodemographic and disability-related factors were associated with this high psychopathology. Attention has to be given by the government and other stakeholders to intervene in psychopathology and its associated factors.

## Statement of the problem

As can be seen from history, different viral diseases happened in the past 20 years in the world. For example, Severe Acute Respiratory Syndrome (SARS) pandemic emerged in 2003, influenza virus outbreaks with the H1N1 subtype in 2009, Middle East Respiratory Syndrome (MERS) in 2012, and the Ebola virus in 2014 [1–3]. The coronavirus is now becoming a serious public health emergency since December 2019 [4]. Symptoms of the Coronavirus infection include fever, chills, cough, sore throat, myalgia, nausea and vomiting, and diarrhea. Individuals with a history of underlying diseases are more likely to be infected with the virus and would experience worse outcomes [5]. Severe cases of the disease can lead to heart, and respiratory failure, acute respiratory syndrome, or even death [6].

Multiple studies showed that such public health emergencies are accompanied by psychopathologies [7, 8]. The coronavirus disease(COVID-19) had also been known to cause great mental health problems in the infected patients, and health care workers, families, children, students, patients with mental illness, and even workers [9–11].

As of February 2020, IASC (Inter-agency standing committee) Reference Group on Mental Health and Psychosocial Support in Emergency Settings developed a guideline. This guideline explained society in general and people with disabilities, in particular, are in fear of being socially excluded/placed in quarantine. The reason for this could be the disease, feeling powerless in protecting loved ones and fear of losing loved ones due to the virus, refusal to care for unaccompanied or separated minors due to fear of infection, because parents or caregivers have been taken into quarantine [12].

A systematic review and meta-analysis on the prevalence of anxiety, depression, and stress among the general population during the COVID-19 revealed that 29.6% of participants had stress, 31.9% had anxiety and 33.7% had depression [13]. Another review and meta-analysis on the mental health impact of COVID-19 on health care professionals reported that anxiety, depression, and anxiety, was prevalent in 23·2%, 22·8%, and 38·9% of the study participants respectively [14]. Multiple earlier studies reported the prevalence of generalized anxiety symptoms, and depressive symptoms among the general population during the COVID-19 period were 35.1%, and 20.1% in China [15], 23.6% and 45.1% in turkey [16], 65% and 69% in Pennsylvania [17].

**Fig 1:**
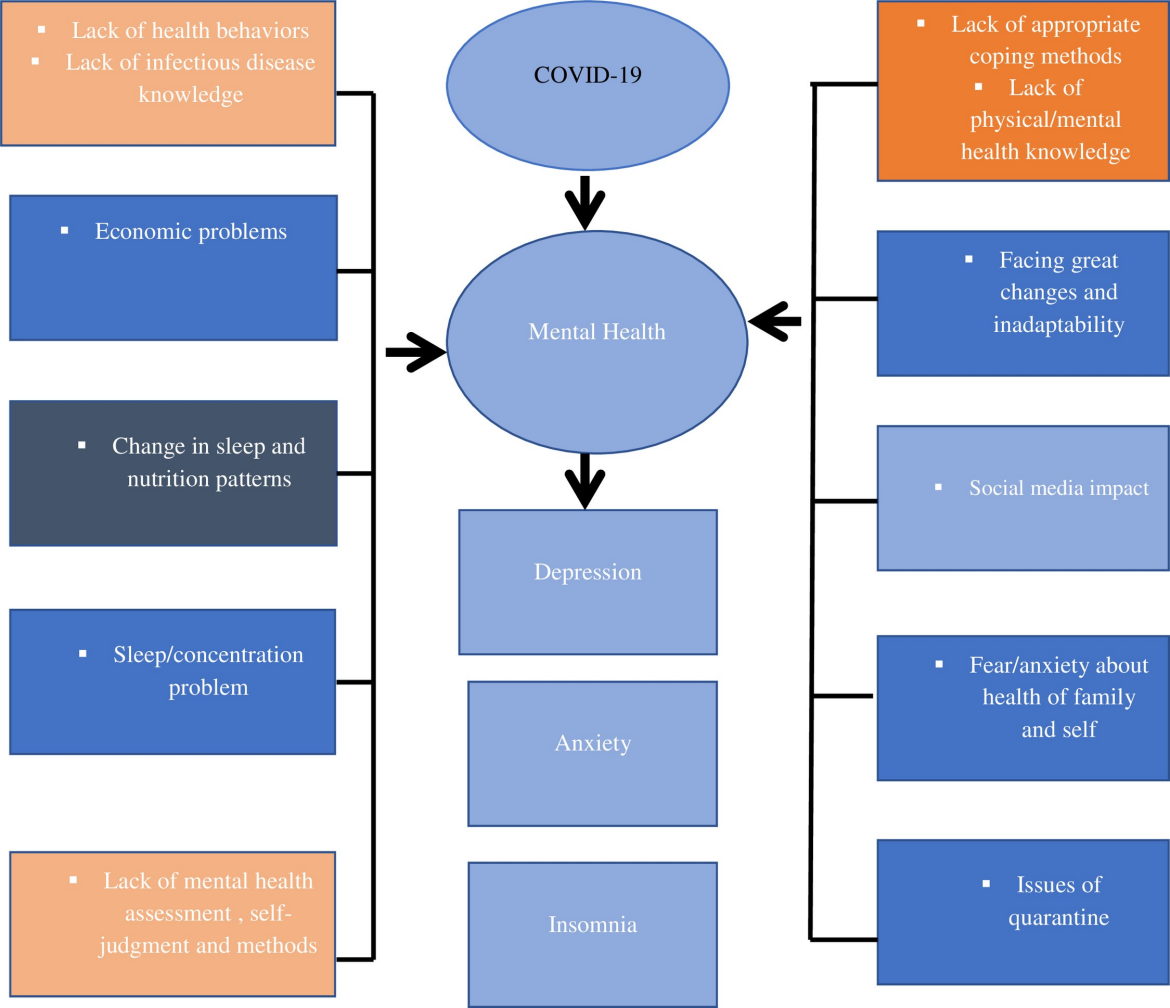
A figure showing the factors that increase the vulnerability to the mental and psychological impact of COVID-19.

Earlier research revealed that attending COVID-19 news frequently [18], misinformation, and fabricated reports about COVID-19 [19], and frequent media exposure may cause distress. Uncertainty regarding one’s health, treatment care, follow-up of patients, and inefficiency increases the vulnerability to the psychological effects of COVID-19 [15, 19–29] (**Fig 1**).

As researchers of the current study, we investigators know that people living with disabilities are subjected to different forms of mental and psychosocial problems in times of emergencies like the COVID-19, and the way they cope with these problems might not be adaptive. Therefore, assessment of the mental and psycho-social needs of people living with a disability during the COVID-19 pandemic will be important in delivering appropriate messages thereby equipping them with updated information, decreasing the risk of infection, and finally improving the mental and psychosocial health of people living with disability in Dessie town.

## Methods and materials

### Ethical approval

Before the beginning of the study, the ethical committee of the college of medicine and health science of Wollo University reviewed the ethical concerns of the study and approved it with a letter (**CMHS-360/013/12**). The participants were given an informed consent sheet before the start of the interview and signed it they were volunteers to proceed in the study. After that, participants who signed the informed consent were interviewed by data collectors and were also having a full right to exit from the interview at any time they want. During the process of interviewing participants, personal identifiers were not used to keep the confidentiality of the collected data.

### Study setting

This cross-sectional survey was done from July 15/2020 to July 30/2020. The study was done in disability association centers in Dessie town. The town is centered in the South Wollo zone of the Amhara region to the North East of Ethiopia. This town is located 401 km far from Addis Ababa; the capital city of Ethiopia and has 18 Kebeles and 350,000 inhabitants based on the latest statistics from the region [30]. There are seven associations of disabled individuals in the town (disability association for individuals living with blindness, disability association for individuals living with hearing impairment, disability association for individuals with mobility limitation, disability association for individuals who are victims of leprosy, disability association for individuals with intellectual disability, disability association for female disabled individuals and disability association for geriatric individuals). More than 1800 individuals with the above types of disability are members of the associations as per the data from the federation for disability in the town.

### Study participants

The sample size for the study was determined using the single population proportion formula and taking the following considerations. 1) The prevalence of depressive symptoms, anxiety symptoms, and insomnia as 50% due to the absence of earlier studies on depression, anxiety, and insomnia in individuals living with disabilities in Ethiopia during the covid-19 pandemic.

Besides, the Zα⁄2-value of 1.96 was used at a 95% Confidence interval and a margin of error of 5%. Finally, a non-response rate of 10% was considered to give a total of 423 people with disabilities planned to be included in the study.

The study was done with a purposive sampling technique to recruit participants from individuals living with various types of disability (blindness, deafness, mobility limitation, and victims of leprosy).

The target populations for the current study were individuals living with various disability types as described above and who are members of the disability association in Dessie town whereas people with disabilities and members of the disability association who avail themselves during the study were considered as the study population. Among the planned 423 participants, we recruited a total of 401 individuals living with disabilities with a complete and consistent response. The remaining participants were excluded from the analysis due to a variety of quality limitations. This makes the response rate of the study to be 94.8.

### Eligibility criteria

The study recruits only individuals living with a disability and members of disability associations whose age was 18 years and older. However, people with intellectual disability and pre-existing known depressive disorder, anxiety disorder, and sleep disturbance were not invited.

### Sociodemographic and clinical characteristics

The sociodemographic characteristics studied and incorporated in the final analysis were age in years (< 25, 25–34, 35–44, 45–54 and ≥ 55), sex (female/male), marital status(married, single and divorced/widowed/separated), ethnicity (Amhara, Oromo, Tigray, and others), religion (orthodox, Muslim, protestant and others), occupation classified as a government-employed, farmer, merchant, housewife, laborer and unemployed, educational status (non-educated, primary school, secondary school, college and above), with whom the individual is living classified as living alone, with family and within a common compound. Besides, clinical variables like concerns of being infected with COVID-19 and risk of infecting others (high, moderate, low and none), and associating the symptoms of other illnesses with the symptoms of COVID-19 (high, moderate, low and none) was measured. Further to this the presence or absence of pre-existing chronic medical illness and type of disability (blindness, deafness, mobility limitation, and victims of leprosy) were evaluated.

### Knowledge regarding COVID-19 and substance-related variables

The knowledge of participants regarding the COVID-19 transmission and epidemiology had been weighed based on the individual response to the specified questions. Besides, substance-related factors such as ever use of substances and current use of substances was measured.

### Measurement of depression

Depression among participants during the COVID-19 pandemic period was screened using the patient health questionnaire-9 (PHQ-9), which consisted of 9-questions adapted to assess the level of depressive symptoms due to the COVID-19 pandemic as ranging from 0(“not at all”) to 3 (“nearly every day”). The sum score of these 9 elements varies from 0 to 27 with a score from 0–4 representing the absence of depression whereas a summed score of 5–9 representing mild depression. A score of 10–14 and 15–19 also defines moderate and moderately severe depression whereas a score higher than 19 represents severe depression. This screening tool was validated in Ethiopia [31] for the screening of depression and major depressive disorder will be diagnosed with a sensitivity of 88% and a specificity of 88% at a cut-off point 10 and above [32].

### Measurement of anxiety

Anxiety during the COVID-19 pandemic was screened using the general anxiety disorder scale (GAD-7) which is a brief 7 item anxiety screening tool developed by Spitzer et al. [33] to assess the presence of GAD in a primary care population. This assessment scale was adapted to evaluate the presence of anxiety in individuals living with a disability. The 7 items are scored from 0 (not at all) to 3 (nearly every day) with an overall GAD-7 scale score ranging from 0 to twenty-one. The scale presents a rapid, efficient, reliable, and valid method for detecting the presence of a common anxiety disorder. A score 0–4 represents minimal anxiety, a score of 5–9: mild anxiety, a score of 10–14: moderate Anxiety, and a score greater than 15: severe Anxiety. A cut-off point at a score of 10 and above on the GAD-7 scale had been defined as GAD with a sensitivity and specificity of 89% and 82% respectively [33].

### Measurement of Insomnia

The presence or absence of sleep disturbance during the COVID-19 pandemic was evaluated with the insomnia severity index [34]. This scale consisted of seven elements that assess the presence of insomnia due to the COVID-19 pandemic. The first three elements (difficulty of falling asleep, the difficulty of staying asleep, problems of waking up too early) assess the severity of insomnia from 0 (none), to 1(mild), 2 (moderate), 3 (severe), and 4(very severe).

The 4^th^ element assesses "how the individual was satisfied/dissatisfied with his/her current sleep pattern" and scored from 0 (very satisfied) to 4(very dissatisfied). The 5^th^ item asses “how noticeable to others do the individual think of his/her sleep problem in terms of impairing quality of life” and rages from 0 (not at all noticeable) to 4 (very much noticeable). The 6^th^ element describes “how worried/distressed was the individual about his/her current sleep problem" and scored from 0 (not at all worried to 4 (very much worried). The final item assesses the extent of interference of sleep problem to the individual daily functioning and varies from 0 (not at all interfering) to 4 (very much interfering). Overall the sum of the 7 items ranges 0–28 with scores of 0–7, 8–14, 15–21, and 22–28 representing no clinically significant insomnia, sub-threshold insomnia, clinical insomnia of moderate severity, and clinical insomnia of severe severity.

### Measurement of coping

Coping of disabled individuals to the psychopathologic impact of the COVID-19 pandemic was assessed using the brief resilient coping scale developed by Sinclair. G., & Wallston, K. A. in 2004 [35]. The scale consisted of four questions (I look for creative ways to alter for difficult situations, Regardless of what happens to me, I believe I can control my reaction to it, I actively look for ways to replace the losses I encounter in life and I believe I can grow in positive ways by dealing with difficult situations). Each of the four items is scored from 1 (does not describe me at all) to 5 (describe me very well). The overall score, therefore, ranges from 4–20 with a score of 4–13 points, 14–16 points, and 17–20 points representing low resilient copers, medium resilient copers, and high resilient copers respectively.

### Data collection procedures and quality control methods

The initial step in the study process was the translation of the English version of questionnaires by both psychiatry and language experts together. The next step was the training of three Bachelor of Science level data collectors on the content and components of data collection tools, ethical issues to be maintained during data collection, the physical distancing, and the use of appropriate respirators during data collection. Then after obtaining ethical clearance from the respected body, a pre-test was done 7 days before the main study at kombolcha town, which is located 15 kilometers far from the study area. After that the data collection for the main study was implemented from July 15/2020 to July 30/2020 using a questionnaire comprised of 1) socio-demographic variables 2) substance use variables 3) the insomnia severity index 4) the patient health questionnaire-9 5) Generalized anxiety disorder-7 6) knowledge regarding transmission and epidemiology of COVID-19 and 7) brief resilient coping scale. During this study period, continuous supervision was conveyed by two Master of Science in mental health professional specialists and timely corrections and support were given to data collectors. Furthermore, the collected questionnaire was checked for clarity and completeness daily and feedback for the next day work was given appropriately. Participants obtained to have serious psychopathology were linked to a nearby psychiatric service center for further management.

### Statistical analysis

After data was entered into EPI-DATA 3.1, it was exported to a statistical software package for social science SPSS V.20 for Windows for analysis. Descriptive statistics like mean and standard deviation (SD) were used to describe continuous data whereas numbers and percentages were employed to describe categorical variables. A binary logistic regression method was used to identify the related factors for the psychopathologies. Furthermore, an odds ratio with its 95%CI was driven to show association strength, and a P-value <0.05 was declared as statistically significant. Model fitness in the multi-variable binary logistic regression was assured with the Hosmer-Lemeshow goddess of fit test and multi-Collinearity diagnosis was done with a standard error.

## Results

### Socio-demographic characteristics of participants

Four hundred twenty-three questionnaires were collected from participants but only four hundred three were found to be complete which makes the response rate to be 95.27%. All participants’ sociodemographic data is indicated in **Table 1**. Of the 403 participants, 239(59.3%) were male, and 164(40.7%) were female. The mean age of participants was 36.66±16.09 years and ranges from 18 to 90 years. When stratified by age, 280(69.5%) of participants are below 40 years and 123(30.5%) were above or equal to 40 years. Nearly half; 207(51.4%) of the study participants were married and one hundred fifty (37.2%) were single. The majority of the study subjects; 363(90.1%) were Amhara in their ethnic background. Considering religion, 199(49.4%) and 163(40.4%) were Orthodox and Muslim followers respectively. Education was categorized into non-educated, the primary school completed, secondary/preparatory school completed, diploma, and above. One hundred fourteen (28.3%) and 108(26.8%) were non-educated and primary school completed respectively. A higher proportion of participants; 169 (41.9%) were unemployed.

**Table 1:** Socio-demographic characteristics of individuals living with a disability.

Characteristics	Category	Value (n, %)
Age in years, mean± SD	36.66±16.09 years	
Age in years	Below 40 years	280(69.5%)
	≥ 40 years	123(30.5%)
Sex	Male	239(59.3%)
	Female	164(40.7%)
Marital status	Married	207(51.4%)
	Single	150(37.2%)
	Widowed /divorced/separated	46(11.5%)
Ethnicity	Amhara	363(90.1%)
	Oromo	29(7.2%)
	Others	11(2.7%)
Religion	Orthodox	199(49.4%)
	Muslim	163(40.4%)
	Protestant	28(6.9%)
	Others*	13(3.2%)
Educational level	Non-educated	114(28.3%)
	Primary school	108(26.8%)
	Secondary &preparatory school	103(25.6%)
	Diploma and above	78 (19.4%)
Employment	Government employed	50 (12.4%)
	Farmer	30 (7.4%)
	Merchant	20 (5.0%)
	Housewife	21 (5.2%)
	Daily Labour	113 (28.0%)
	Unemployed	169 (41.9%)
With whom you live	Alone	183(45.4%)
	With family	210(52.1%)
	With friends	10(2.5%)

### Clinical, COVID-19 and substance-related characteristics of individuals living with a disability

Of all study participants; 319(78.9%) worried about being infected with COVID-19 or transmitting it to other family members, 227(56.3%) had a high level of fear for oneself and one’s family getting infected, 208(51.6%) associates the symptoms of other illness with that of the coronavirus disease. Regarding the knowledge of participants to COVID-19; 339(84.1%) believe that COVID-19 is transmitted by droplets, 297(73.7%) believed that COVID-19 is an airborne disease, and 345(85.6%) believed that COVID-19 is transmitted by contaminated objects. One hundred seven (26.6%) of the study subjects have a diagnosed medical illness but 296(73.4%) were not diagnosed to have a medical illness. The 403 participants with a disability include blindness 81(20.1%), deafness 93 (23.1%), leprosy victims 74 (18.4%), and mobility limitation 155(38.4%). Details regarding Clinical, COVID-19, and substance-related characteristics of participants are indicated **in Table 2.**

**Table 2:** Clinical, COVID-19, and substance-related characteristics of individuals living with a disability.

Characteristics	Category	Value (n, %)
Worried about being infected with COVID-19 or transmitting it to other family members	Yes	319(78.9%)
	No	85(21.1%)
Level of fear for oneself and one’s family getting infected	High	227(56.3%)
	Moderate	132(32.8%)
	Mild	44(10.9%)
Level of worry about getting infected when showing symptoms associated with Novel Coronavirus Pneumonia (NCP)	High	208(51.6%)
	Moderate	147(36.5%)
	Low	31(7.7%)
	None	17(4.2%)
Believe that covid-19 is transmitted by droplets	Yes	339(84.1%)
	No	49(12.2%)
	Uncertain	15(3.7%)
Believe that covid-19 is an airborne disease	Yes	297(73.7%)
	No	96(23.8%)
	Uncertain	10(2.5%)
Believe that covid-19 is transmitted by contaminated objects	Yes	345(85.6%)
	No	52(12.9%)
	Uncertain	6(1.5%)
Know that the number of covid-19 cases is increasing	Yes	314(77.9%)
	No	89(22%)
Know that the number of covid-19 related deaths is increasing	Yes	340(84.4%)
	No	58(14.6%)
	Uncertain	4(1.0%)
Chronic comorbid disease	Yes	107(26.6%)
	No	296(73.4%)
Type of disability	Blindness	81(20.1%)
	Deafness	93(23.1%)
	Leprosy victims	74(18.4%)
	Mobility limitation	155(38.4%)
Ever substance use	Yes	118(29.3%)
	No	285(70.7%)
Type of ever substance use	Alcohol	51(12.7%)
	Cigarette	47(11.7%)
	Khat	36(8.9%)
	None	269(66.7%)
Current substance use	Yes	103(25.6%)
	No	300(74.4%)
Type of current substance use	Alcohol	58(14.4%)
	Cigarette	58(14.4%)
	Khat	39(9.6%)
	None	248(61.5%)

### Prevalence of depression, anxiety, insomnia, and coping mechanisms of individuals living with a disability

**Fig 2:**
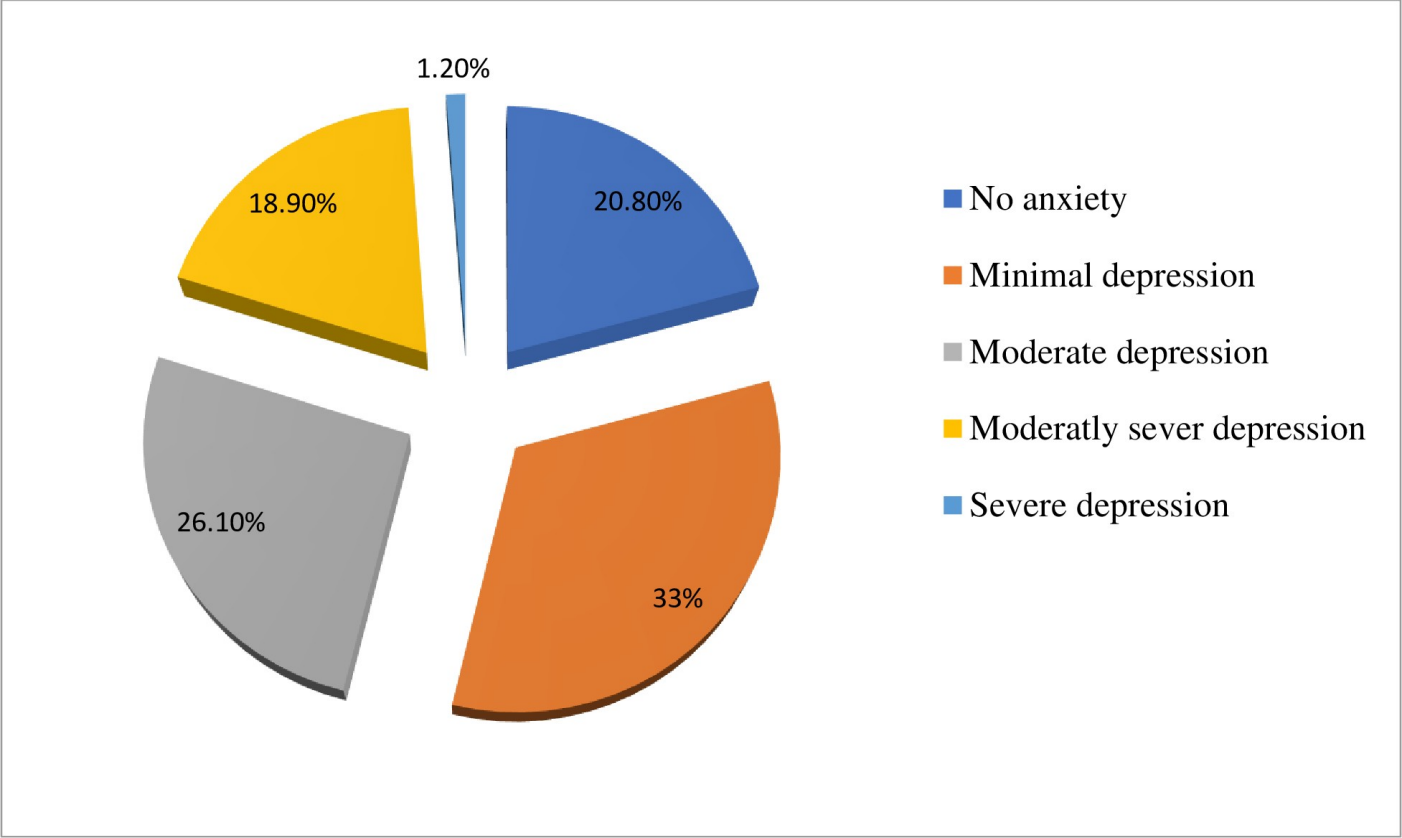
A chart showing the severity levels of depressive symptoms among study participants in the study.

**Fig 3:**
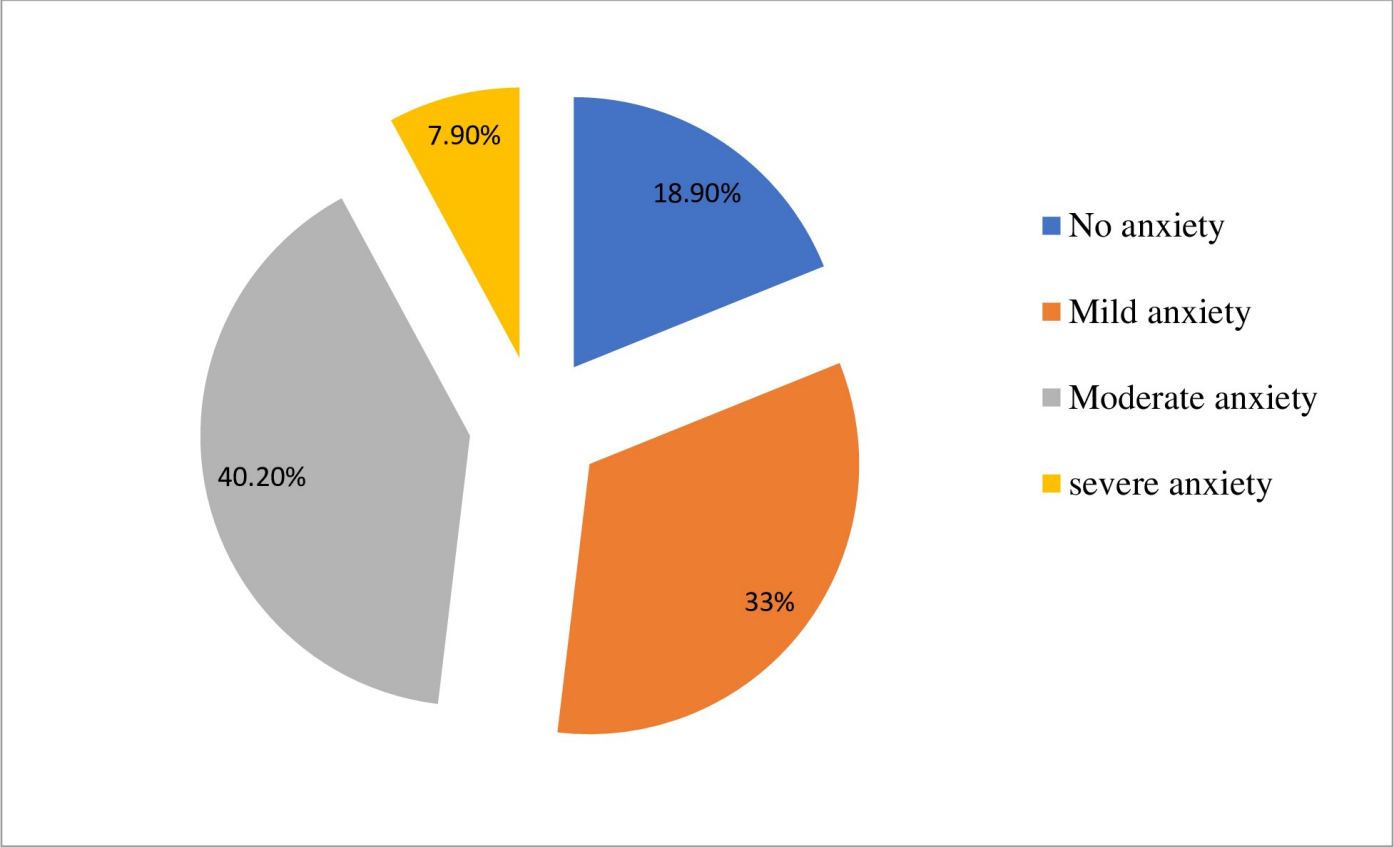
A chart showing the severity levels of anxiety symptoms among study participants in the study.

**Fig 4:**
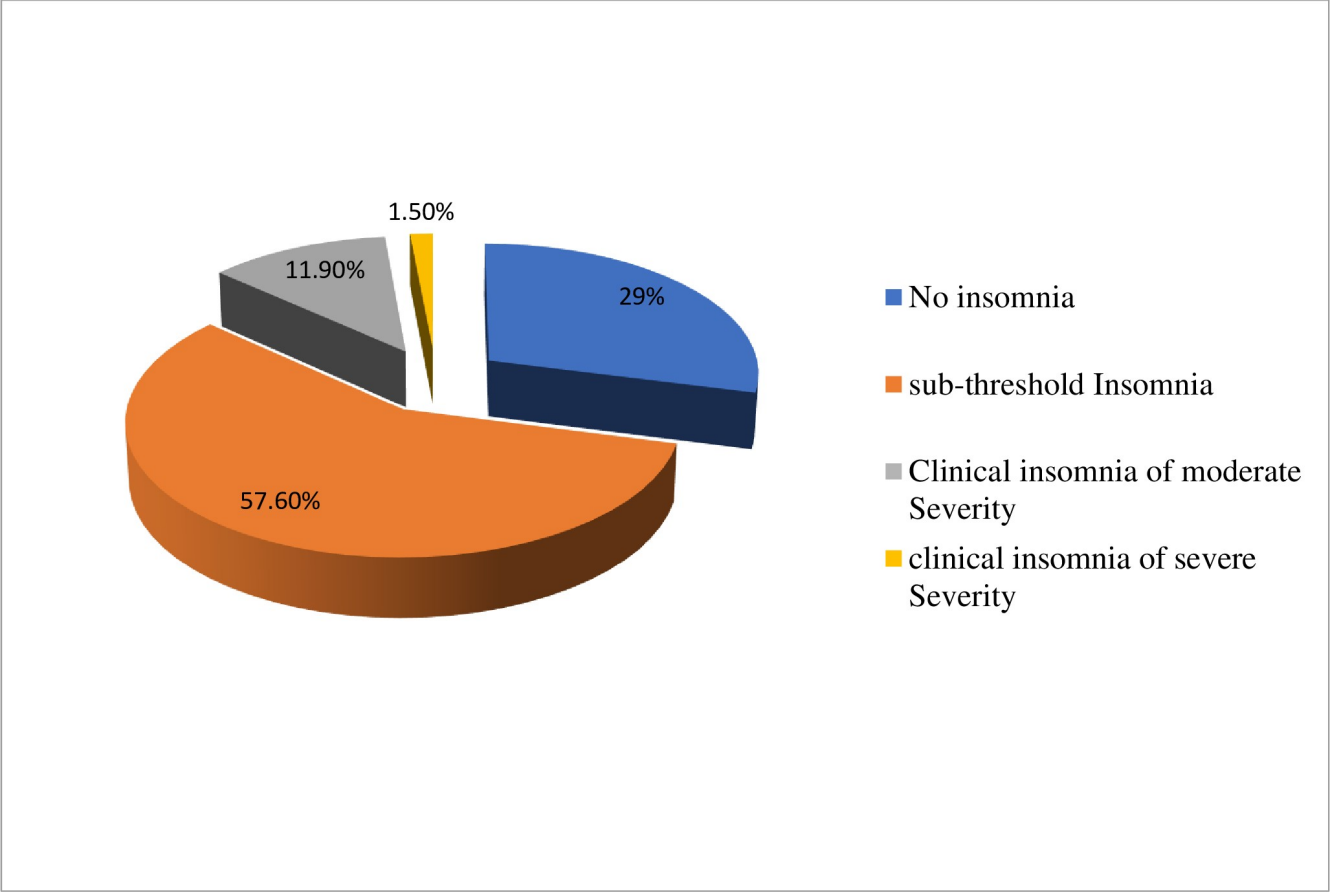
A chart showing the severity levels of insomnia symptoms among study participants in the study.

The overall prevalence of depression and anxiety among the study participants was 186(46.2%) and 194(48.1%) respectively at a cut of points of 10 and above on both the PHQ-9 depression scale and the GAD-7 anxiety scale. Considering severity, 33%, 26.1%, 18.9%, and 1.2% of study subjects were found to have mild, moderate, moderately severe, and severe depression respectively **(Fig 2).** On the other hand, 33%, 40.2%, and 7.9% of participants had mild, moderate, and severe anxiety (**Fig 3**). Participants who were not educated have a higher proportion of depression (57.9%) than participants who were a diploma and above (37.2%). Females have a relatively higher prevalence of anxiety (53%) than males (44.8%) however this was not valid statistically (P-value = 0.25). widowed/divorced and separated individuals had a higher proportion of depression (67.4% VS 41%), anxiety (58.5% VS 41.5%) than married participants. Considering the type of disability, depression was highest; 64 (86.5%) in victims of leprosy, and this was also supported statistically (**AOR = 9.2; 95% CI: 4.93, 21.21**), anxiety was highest in individuals with deafness; 80 (86%). This was also significant statistically (**AOR = 8.2; 95% CI: 4.52, 19.43%).** Insomnia was found in 280 (71%) of study participants. However, its severity varies and 232 (57.6%), 48 (11.9%), and 6 (1.1%) of participants had sub-threshold, moderate, and severe insomnia respectively **(Fig 4)**. Regarding coping, 45.7%, 37.5%, and 16.9% of participants were low, moderate, and high resilient copers respectively. Detailed information on the prevalence of anxiety, depression, insomnia, and coping are shown **in Table 3** below.

**Table 3:** Prevalence of depression, anxiety, insomnia and coping by different characteristics.

Parameter	Category	Depression, n (%)	Anxiety symptom, n(%)	Insomnia			Coping mechanism		
				Sub threshold, n (%)	Moderate, n (%)	Severe, n (%)	Low resilient copers, n (%)	Moderate resilient copers, n (%)	High resilient copers n (%)
Age in years	< 40 years	100(35.7%)	148(52.%)	161(57.5%)	26 (9.3%)	3(1%)	116(41.4%)	111(39.%)	53(18.9%)
	≥ 40 years	86 (69.9%)	46(37.4%)	71 (57.72%)	22(17.88%)	3(2.44%)	68(55.3%)	40(32.5%)	15(12.2%)
Sex	Male	113(47.3%)	107(44.%)	131(54.8%)	33 (13.8%)	3(1.25)	115(48.1%)	82(34.3%)	42(17.6%)
	Female	73(44.5%)	87(53%)	101(61.6%)	15(9.1%)	3(1.8%)	69(42%)	69 (42%)	26(15.8%)
Marital status	Married	85(41%)	86(41.5%)	106(51.2%)	24(11.6%)	2(0.96%)	95(45.9%)	68(32.8%)	44(21.2%)
	Single	70(46.7%)	81(54%)	95(63.3%)	17(11.3%)	3(2%)	68(45.3%)	67(44.7%)	15(10%)
	Widowed/divorced/separated	31(67.4%)	27(58.5%)	31(67.4%)	7(15.2%)	1(2.2%)	21(45.6%)	16(34.8%)	9(19.6%)
Education	Non educated	66(57.9%)	47(41.2%)	75(65.8%)	14(12.3%)	4(3.5%)	62(54.4%)	36(31.6%)	16(14%)
	1ry school	53(49%)	52(48.1%)	58(53.7%)	17(15.7%)	0(0%)	51(47.2%)	46(42.6%)	11(10.2%)
	2ry&preparatory school	38(36.9%)	67(65%)	53(51.5%)	12(11.6%)	0(%)	43(41.7%)	32(31%)	28(27.2%)
	Diploma and above	29(37.2%)	28(35.9%)	46(58.9%)	5(6.4%)	2(2.3%)	28(35.9%)	37(47.4%)	13(16.7%)
Employment	Govtemploye	15(30%)	14(28%)	25(50%)	2(4%)	0(0%)	27(54%)	17(34%)	6(12%)
	Farmer	15(50%)	20(66.7%)	17(56.7%)	1(3.33%)	0(0%)	15(50%)	4(13.33%)	11(36.7%)
	Merchant	5(25%)	6(30%)	11(55%)	1(5%)	1(5%)	10(50%)	7(35%)	3(15%)
	Housewife	7(33.3%)	9(42.8%)	11(52.4%)	2(9.5%)	2(9.5%)	7(33.33%)	12(57%)	2(9.5%)
	Daily Labour	60(53%)	61(53.9%)	60(53%)	21(18.6%)	2(1.7%)	51(45.1%)	41(36.3%)	21(18.6%)
	Unemployed	84(49.7%)	84(49.7%)	108(63.9%)	21(12.4%)	1(0.6%)	74(43.8%)	70(41.4%)	25(14.8%)
Comorbid illness	Yes	49(45.8%)	60(56%)	63(58.9%)	13(12.2%)	1(0.93%)	56(52.3%)	44(41.1%)	7 (6.5%)
	No	137(46.3%)	134(45.3%)	169(57%)	35(11.82%)	5(1.7%)	128(43.2%)	107(36.%)	61(20.6%)
Disability type	Blindness	31(38.3%)	33(40.7%)	55(67.9%)	3(3.7%)	1(1.23%)	40(49.4%)	40(49.4%)	1(1.23%)
	Deafness	34(36.6%)	80(86%)	58(62.4%)	24(25.8%)	1(1.07%)	37(39.8%)	43(46.2%)	13(13.9%)
	Leprosy victims	64(86.5%)	24(32.4%)	47(63.5%)	17(22.9%)	4(5.4%)	42 (56.7%)	20(27%)	12(16.2%)
	Mobility limitation	57(36.7%)	57(36.8%)	72(46.5%)	4(2.6%)	0(0%)	65(41.9%)	48(30.9%)	42(27%)

### Associated factors for depression, anxiety, and insomnia among study participants

We used the binary logistic regression analysis method to identify the associated factors for anxiety, depression and insomnia. Independent variables with p-value <0.025 in the bi-variate analysis for anxiety, depression and insomnia were fitted to the multivariate logistic regression. At the multivariate analysis we used p-value <0.05 to identify associated factor for the outcome variables.

The result of final model revealed that individuals with disability who were divorced/widowed/separated were significantly more likely to have depression (AOR = 3.4, 95% CI: 1.28–8.92, P-value = 0.006) than married counterparts. Depression was also significantly associated with educational status (non-educated) (AOR = 2.12, 95% CI: 1.12, 5.90, P-value = 0.001), and unemployment (AOR = 2.1, 95% CI: 1.32, 5.11, P-value = 0.005) as well as being a daily laborer (AOR = 2.4, 95% CI: 1.20, 4.89, P-value = 0.014).

Generalized anxiety disorder was significantly higher in young age (<40 years) (AOR = 1.7, 95% CI: 1.32, 2.98, P-value = 0.02). Moreover, anxiety was significantly higher in single (AOR = 2.3, 95% CI: 1.24, 5.3, P-value = 0.011) and widowed/divorced/separated (AOR = 1.5, 95% CI: 1.12, 2.78, P-value = 0.032) participants than married ones. Besides, anxiety was higher in preparatory school completed (AOR = 3.00, 95% CI: 1.59, 5.46, P-value = 0.001), daily laborer (AOR = 2.7, 95% CI: 1.21, 5.23, P-value = 0.003), unemployed (AOR = 2.5, 95% CI: 1.17, 4.78, P-value = 0.005) and individual with deafness (AOR = 8.2, 95% CI: 4.52, 19.43, P-value = 0.00) at a significant level.

Moreover, insomnia was significantly higher in single (AOR = 1.5, 95% CI: 1.12, 3.09, P-value = 0.027), divorced/widowed/separated(AOR = 6.2, 95% CI: 1.08, 11.29, P-value = 0.032), unemployed (AOR = 3.00, 95% CI: 1.22, 7.03, P-value = 0.001), blind (AOR = 2.8, 95% CI: 1.42, 6.35, P-value = 0.001), deaf (AOR = 10.2, 95% CI: 4.52, 35.33, P-value = 0.002) and leprosy victim (AOR = 21.4, 95% CI: 4.03, 32.65, P-value = 0.001) participants as compared to their reference groups. Details concerning the associated factors for anxiety, depression and insomnia are presented in **Table 4.**

**Table 4:** Factors associated with depression symptom, anxiety symptom, and Insomnia in individuals with a disability during the COVID-19 period.

Parameters	Category	Depression symptom			Anxiety symptom			Insomnia		
		AOR	95% CI	P-value	AOR	95% CI	P-value	AOR	95% CI	P-value
Age in years	< 40 years	0.21	0.12, 0.62	**0.00**	1.7	1.32,2.98	**0.02**	0.41	0.24, 0.89	**0.025**
	≥ 40 years	**1.00**			**1.00**			**1.00**		
Sex	Male	**1.00**			**1.00**			**1.00**		
	Female	1.02	0.67, 2.34		1.2	0.9, 2.95		1.3	0.67, 2.15	
Marital status	Married	**1.00**			**1.00**			**1.00**		
	Single	1.11	0.85, 2.78		2.3	1.24, 5.3	**0.011**	1.5	1.12, 3.09	**0.027**
	Widowed/divorced/separated	3.4	1.28, 8.92	**0.006**	1.5	1.12, 2.78	**0.032**	6.2	1.08, 11.29	**0.032**
Education	Non-educated	2.12	1.32, 5.11	**0.005**	1.13	0.59, 2.32		1.20	0.67, 2.38	
	1ry school	1.26	0.75, 4.24		1.58	1.09, 3.15)		0.90	0.52, 2.19	
	2ry&preparatory school	0.76	0.26, 2.90		3.00	1.59, 5.46	**0.001**	0.45	0.40, 1.13	
	Diploma and above	**1.00**			**1.00**			**1.00**		
Occupation	Govt employed	**1.00**			**1.00**			**1.00**		
	Farmer	1.8	0.86, 4.21		3.34	1.39,8.12	**0.002**	0.9	0.28, 1.72	
	Merchant	0.75	0.23, 2.83		0.91	0.32, 2.56		1.02	0.81, 2.32	
	Housewife	1.1	0.67, 2.59		1.52	0.87, 4,79		2.41	0.81, 5.47	
	Daily Labour	2.4	1.12, 5.90	**0.001**	2.7	1.21, 5.23	**0.003**	1.3	0.97, 3.10	
	Unemployed	2.1	1.20, 4.89	**0.014**	2.5	1.17, 4.78	**0.005**	3.0	1.22, 7.03	**0.001**
Comorbid illness	Yes		0.84	0.58, 1.46	1.43	0.54, 4.21		0.89	0.48, 1.56	
	No	**1.00**			**1.00**			**1.00**		
Disability type	Blindness	0.82	0.44, 1,62		1.21	0.56, 1.84		2.8	1.42, 6.35	**0.001**
	Deafness	0.35	0.12, 1.55		8.2	4.52, 19.43	**0.00**	10.2	4.52, 35.33	**0.002**
	Leprosy victims	9.2	4.93, 21.21	**0.00**	0.6	0.29, 1.88		21.4	4.03, 32.65	**0.001**
	Mobility limitation	**1.00**			**1.00**			**1.00**		

## Discussion

Per the knowledge of researchers of the current study, this work is the first study that assessed the mental and psychological impact of COVID-19 on individuals living with disabilities. The COVID-19 pandemic, with its rapid transmission rate, and the high fatality rate has intensified anxiety among the global population, leading to psychological and mental disorders in the global community. Moreover, stereotyping and discrimination are rampant in these populations at this period [36, 37].

It is therefore essential to study and distinguish the mental and psychological states of individuals in this unprecedented, perplexing, and critical time. Earlier evidence suggests that suicidal thoughts, psychosis, anxiety, trauma, and panic attacks are common in this time [18, 38]. Other evidence also suggested that COVID-19 had a high degree of psychological outcomes such as depression, anxiety, and post-traumatic stress symptoms [21, 22, 26] due to its rapid transmission, its high mortality rate, and concerns about the future [39]. Anxiety had also an impact on the body's immune system and so that intensifying the risk of contracting the virus [18].

Therefore, the result obtained from this study on the prevalence of anxiety, depression, insomnia, and coping style of people living with a disability will be significant evidence to policymakers and program planners' to initiate a recommendation for early initiation of interventions. Furthermore, clinicians, further researchers, and administrative bodies will use the evidence generated from the current study as a baseline for further investigation.

This result of this study revealed that the prevalence of depression, anxiety, and insomnia in individuals with a disability in response to the pandemic is 46.2%, 48.1%, and 71% respectively, and found that single, divorced/widowed/separated, non-educated, daily labor and unemployed participants, deafness and leprosy victim types of disability were more severely affected.

This study revealed that the young age group (<40 years) increases the risk of anxiety in participants. Individuals who were less than 40 years of age were 1.7 times more likely to have anxiety than older age groups (≥40 years). The reason for this could be individuals in the young age group are more troubled over the future impacts and economic encounters due to the pandemic, since they are the most productive forces in the society [15, 20, 21]. Moreover, the higher rate of exposure to social media concerning the transmission, and case fatality rate might increase the stress and anxiety [40].

Marital status was found to be significantly associated with anxiety, depression, and insomnia in the present study. Individuals with a disability who were divorced/widowed/separated and single were significantly more likely to have depression, anxiety, and insomnia than married counterparts. Single participants are emotionally more damaged by stress full experience than the married participants [41] that would heathen the depression and anxiety.

In the present study individuals with a low level of education were significantly at a higher level of depression and anxiety than individuals with a higher level of education. This was, however, in contradiction to a study finding in earlier studies. Studies in Iran and china revealed that individuals at a higher level of education were more increasingly vulnerable to anxiety and depression to the impacts of COVID-19 [20, 26]. The level of self-awareness of one's own perceived health in individuals with a high level of education could be the cause [42]. Also, the high number of significant networks in these individuals will cause anxiety and depression [20, 22, 43].

Unemployment is a risk factor for depression and anxiety. In the present study too, individuals who were unemployed and daily laborer were more likely affected by depression, anxiety, and insomnia than the government employed. This was consistent with multiple earlier evidence [44–46]. Disappointed hope and financial problems which are the daily hassles of unemployment, might have a role in this association [47].

Although not the case in the present study, epidemiological evidence revealed that women are more vulnerable to anxiety and depression than men [48]. The high vulnerability to stress and post-traumatic stress disorder in women could be responsible for this [20, 49]. This was supported by a recent study on the prevalence of depression symptoms, anxiety symptoms during the COVID-19 pandemic [19, 20, 23, 26].

Moreover, the presence of a comorbid medical illness was not found to be significantly associated with depression, anxiety, and insomnia in the present study. This was, however, in opposition to the result of a study on the psychosocial impact of COVID-19 in Italy [29] that obtained a significant correlation between medical illness and anxiety/depression. The high sense of vulnerability to COVID-19 in individuals with earlier medical and psychiatric illness could be responsible for this [50].

## Conclusion

This study revealed that depression, anxiety, and insomnia were highly prevalent in individuals living with a disability during the COVID-19 pandemic. It was also found that single, divorced/widowed/separated, non-educated, daily labor and unemployed participants, deafness, and leprosy victim types of disability were more severely affected by depression and insomnia. This implies the need for recognition of this target population for psychosocial support and interventions during the COVID-19 pandemic. Moreover, the above mentioned socio-demographic profiles need to be considered during the designing and implementation of psychosocial interventions. Furthermore, binding procedures and guidelines have also to be developed and used.

## Implications of the study

The major implication of this study is the high occurrence of anxiety symptoms, depression symptoms, insomnia, and identification of the degree of coping among individuals living with disabilities due to the covid-19 pandemic in Dessie town. The result of this study is the first of its kind in the target population recommends the necessity of psychosocial interventional efforts to advance screening and management of anxiety symptoms, depressive symptoms, and insomnia due to the Covid-19 pandemic. This benefits the unreached target population in terms of reversing the psychosocial burden of Covid-19. Moreover, it is imperative to have an improved understanding of depression symptoms, anxiety symptoms, insomnia, and integrate these domains during routine service delivery.

## Supporting information

S1 Questionnaire English version of the questionnaire. (DOCX)

S2 Questionnaire Amharic version of the questionnaire. (DOCX)

## List of Abbreviations

AOR: Adjusted Odds Ratio
CI: Confidence Interval
COVID-19: Coronavirus disease-19
GAD-7: Generalized Anxiety Disorder-7
PHQ-9: Patient Health Questionnaire-9
SD: Standard Deviation
