# Supplementary material for: Depression, anxiety symptoms, Insomnia, and coping during the COVID-19 pandemic period among individuals living with disabilities in Ethiopia, 2020
Source: PLoS One. 2020 Dec 30;15(12):e0244530. doi: 10.1371/journal.pone.0244530 (PMC7773255; doi:10.1371/journal.pone.0244530)
Supplement: S1 Questionnaire — (DOCX) [file pone.0244530.s001.docx]

**Wollo University, college of medicine and health science, department of community and mental health nursing**

**Section-1**: Socio –demographic variables related to health professionals

Please read the following questions and circle your choice or fill in the blank space

| NO | Question | Response |
| --- | --- | --- |
| 101 | How old are you? | ------------ years |
| 102 | What is your sex? | 1. Male 2. Female |
| 103 | What is your marital status? | 1. Married 2. Single  3. Divorced 4. Widowed 5. Separated |
| 104 | What is your ethnicity? | 1. Amhara 2. Oromo  3. Tigrigna 4. Other(Specify)________ |
| 105 | What is your religion? | 1. Orthodox 2. Muslim  3. Protestant 3. Other(Specify)_________ |
| 106 | What is your educational status? | 1. College diploma 2. BSc degree  3. MSc degree 4. General physician  5. Specialist |
| 107 | Employment status? | 1. Government employed 2. Farmer 3. Merchant 4. House wife 5. Daily laborer 6. Others |
| 108 | With whom the individual is living? | 1. Alone 2. With family 3. Others |
| 109 | How many family members live in the house? | 1. Six people or more 2. three to five people  3. Two and one |
| 110 | Do you afraid that covid-19 will infect my family and me | 1. Yes 2. No |
| 111 | If your answer is yes to 110,What is the level of you fear | 1. High 2. Moderate 3. low |
| 112 | Do you associate the symptoms of other illness like pneumonia with the symptoms of covid-19 | 1. High 2. Moderate 3. Low 4. None |
| 113 | Do you have comorbid medical illness? | 1. Yes 2. No |
| 114 | Type of disality | -------------------------------------- |

**Section-2: the following questions assess the depression symptoms caused by COVID-19. Please** read the following questions on the left and mark ‘‘√ ’’ on the answer that best describe how you have been feeling over the past 2 weeks. Mark only one answer for each question.

Over the last 2 weeks, how often have you been bothered by any of the following problems?

| No | Questions item | Possible Answers | | | |
| --- | --- | --- | --- | --- | --- |
|  |  | Not at all | Several days | More than half the days | Nearly every day |
| 401. | Little interest or pleasure in doing things |  |  |  |  |
| 402 | Feeling down, depressed, or hopeless |  |  |  |  |
| 403 | Trouble falling/staying asleep, sleeping too much |  |  |  |  |
| 404 | Feeling tired or having little energy |  |  |  |  |
| 405 | Poor appetite or overeating |  |  |  |  |
| 406 | Feeling bad about yourself or that you are a failure or have let yourself or your family down |  |  |  |  |
| 407 | Trouble concentrating on things, such as reading the newspaper or watching television. |  |  |  |  |
| 408 | Moving or speaking so slowly that other people could have noticed. Or the opposite; being so fidgety or restless that you have been moving around a lot more than usual. |  |  |  |  |
| 409 | Thoughts that you would be better off dead or of hurting yourself in some way |  |  |  |  |

**Section-3: the following questions assess the anxiety symptoms caused by COVID-19. Please** read the following questions on the left and mark ‘‘√ ’’ on the answer that best describe how you have been feeling over the past 2 weeks. Mark only one answer for each question.

| **No** | **Questions** | **Possible Answers** | | | |
| --- | --- | --- | --- | --- | --- |
|  |  | Not at all | Several days | Over half of the day | Nearly everyday |
| 301 | How often you Feel nervous, Anxious or on edge |  |  |  |  |
| 302 | How often you Feel Not being able to stop or control worrying |  |  |  |  |
| 303 | How often you Feel Worrying too much about different things |  |  |  |  |
| 304 | How often you Feel Trouble relaxing |  |  |  |  |
| 305 | How often you Feel Being so restless that it’s hard to sit still |  |  |  |  |
| 306 | How often you Feel Becoming easily irritable or annoyed |  |  |  |  |
| 307 | How often you Feel Feeling afraid as if something awful might happen |  |  |  |  |

**Section** 4**:** A**ssessment of knowledge of health care professionals regarding covid-19**

| No | Questions |  |
| --- | --- | --- |
| 701 | Do you agree that covid-19 is transmitted with droplets? | 1. Yes 2. No 3. I do not know |
| 702 | Do you agree that covid-19 is air borne? | 1. Yes 2. No |
| 703 | Do you agree that covid-19 is transmitted via contaminated objects? | 1. Yes 2. No 3. I do not know |
| 704 | Have you heard that the number of infected COVID-19 individuals has increased? | 1. Yes 2. No |
| 705 | Have you heard that the number of COVID-19 deaths has increased? | 1. Yes 2. No |

**Section 5: substance use assessment questionnaire**

| **No** | **Questions** |  |
| --- | --- | --- |
| **701** | Have you ever used any substance? | 1. **Yes 2. No** |
| **702** | If your answer to the above question is yes, which type of substance do you use? | **Specify ---------------------------------------------------------------** |
| **703** | Have you ever used any substance in the past 1 month? | 1. **Yes 2. No** |
| **704** | If your answer to the above question is yes, which type of substance do you use? | **Specify ---------------------------------------------------------------** |

**Section -**6: Insomnia severity index questionnaire

| No | Question | Answer to questions | | |  |  |
| --- | --- | --- | --- | --- | --- | --- |
|  |  |  |  |  |  |  |
|  |  | None | Mild | Moderate | Severe | Very severe |
| 1 | Difficulty falling asleep | 0 | 1 | 2 | 3 | 4 |
| 2 | Difficulty staying asleep | 0 | 1 | 2 | 3 | 4 |
| 3 | Problems waking up too early | 0 | 1 | 2 | 3 | 4 |
| 4 | How satisfied/dissatisfied are you with your sleep pattern? | Very Satisfied | Satisfied | Moderately Satisfied | Dissatisfied | Very Dissatisfied |
| 5 | How noticeable to others do you think your sleep problem is in terms of impairing the quality of your life? | Not at all  Noticeable | A Little | Somewhat | Much | Very Much Noticeable |
| 6 | How worried/distress are you about your current sleep problem? | Not at all  Worried | A Little | Somewhat | Much | Very Much Worried |
| 7 | To what extent do you consider your sleep problem to interfere with your daily functioning (e.g. daytime  Fatigue, mood, ability to function at work/daily chores, concentration, memory, mood, etc.) Currently? | Not at all  Interfering | A Little | Somewhat | Much | Very Much Interfering |

**Section-7:** the following questions assess the **coping strategies** of health professionals for psychological distress, depression and anxiety caused by **COVID-19 using the Brief resilient coping scale.** Please read the following questions on the left and mark ‘‘√ ’’ on the answer that best describe your choice. Only one answer is possible.

| **No**     \|  \| \| --- \| | **Question** | **Possible Answers** | | | | | |
| --- | --- | --- | --- | --- | --- | --- | --- | --- |
|  |  | **Does not describe me at all** | **Does not describe me** | **Neutral** | **Describes me** | **Describes me very well** |  |
| **601** | I look for creative ways to alter for difficult situations |  |  |  |  |  |  |
| **602** | Regardless of what happens  to me, I believe I can control  my reaction to it. |  |  |  |  |  |  |
| **603** | I actively look for ways to  replace the losses  I encounter in life. |  |  |  |  |  |  |
| **604** | I believe I can grow in  positive ways by dealing  With difficult situations. |  |  |  |  |  |  |
