# Supplementary material for: Depression, anxiety symptoms, Insomnia, and coping during the COVID-19 pandemic period among individuals living with disabilities in Ethiopia, 2020
Source: PLoS One. 2020 Dec 30;15(12):e0244530. doi: 10.1371/journal.pone.0244530 (PMC7773255; doi:10.1371/journal.pone.0244530)
Supplement: S2 Questionnaire — (DOCX) [file pone.0244530.s002.docx]

**አማርኛ ትርጉም መጠይቅ**

**ወሎ ዩኒቨርሲቲ የሕክምና እና የጤና ሳይንስ ኮሌጅ፣ የስነ-አእምሮ ጤና ነርሲንግ ትምህርት ክፍል**

**የአማርኛ ቃለመጠይቅ ስምምነት**

ቀን ______ / _____ / _____

ውድ ተሳታፊ! ስሜ __________________________ነው ፡፡ አሁን በተከሰተው የኮሮና ቫይረስ ወቅት በደቡብ ወሎ በደሴ ከተማ የሚኖሩት አካል ጉዳተኞች የአእምሮ ጤና ሁኔታን እና ተጓዳኝ ጉዳዮችን ለማወቅ **በ ሞገሴ ነ ጮ** በሚደረገው ጥናት መረጃ ሰብሳቢ ነኝ ፡፡

አሁን በዚህ ምርምር ለመሳተፍ ፈቃደኛነትዎን በሙሉ ፍላጎትዎ ልጠይቅዎት እፈልጋለሁ ፡፡

በጥናቱ ለመሳተፍ ፈቃደኛ ነዎት? ሀ. አዎ ለ. አይደለሁም

መጠይቁን ያስሞላው ሰው ስም ----------------------------ፊርማ------------ቀን------ወር-------ዓ.ም------

የተቆጣጣሪ ስም---------------------------------------------ፊርማ------------ቀን------ወር--------ዓ.ም----

**ክፍል 1:- የታካሚውን የስነ-ልቦና ስነ-ሕዝብ ባህሪያትን የሚመለከቱ ጥያቄዎች**

| ኮድ | ጥያቄዎች | መልስ |  |
| --- | --- | --- | --- |
| ጥ101 | ዕድሜ | ________ዓመት |  |
| ጥ102 | ፆታ | 1.ወንድ 2. ሴት |  |
| ጥ103 | የጋብቻ ሁኔታ | 1.ያገባ/ች 2. ያላገባ/ች  3.የፈታ/ች 4.የሞተበት/ባት |  |
| ጥ104 | ብሔር | 1.አማራ 2. ኦሮሞ  3.ትግሬ 4.ሌላ ________ |  |
| ጥ105 | ሀይማኖት | 1.ኦርቶዶክስ 2.ሙስሊም  3. ፕሮቴስታንት 4. ሌላ |  |
| ጥ106 | የትምህርት ደረጃ | 1.ያልተማረ/ች 2.1ኛ ደረጃ  3.2ኛ ደረጃ 4.ከዚያ በላይ |  |
| ጥ108 | የስራ ሁኔታ | 1.የመንግስት ሰራተኛ 2.ገበሬ  3. ነጋዴ 4. የቤት እመቤት  5.የቀን ሰራተኛ 6.ሌላ _____ |  |
| ጥ109 | አሁን ከማን ጋር ነው የሚኖሩት? | 1. ብቻየን 2. ከቤተሰብ ጋር  3. በጋራ አፓርትመንት ውስጥ |  |
| ጥ110 | በ COVID-19 እያዛለሁ ወይም ወደ ሌሎች የቤተሰብ አባላት ይተላለፋል ብለው በጣም ተጨንቀዋል? | 1. አዎ 2. የለም |  |
| ጥ111 | በበሽታው እያዛለሁ ወይም ወደሌሎች የቤተሰብ አባላት ይተላለፋል ብሎ የመፍራት ደረጃ እንዴት ነው? | 1. ከፍተኛ 2. መካከለኛ  3. ዝቅተኛ 4. የለም |  |
| ጥ112 | ከኖቭል ኮሮና ቫይረስ የሳንባ ምች ጋር ተያይዘው የሚመጡ ምልክቶች በሚታዩበት ጊዜ የበሽታው ምልክት ነው ብሎ የመጨነቅ ደረጃው እንዴት ነው? | 1. ከፍተኛ 2. መካከለኛ  3. መካከለኛ 4. የለም |  |
| ጥ113 | ሥር የሰደደ በሽታ አለብዎት? | 1.አዎ 2. የለም |  |
| ጥ114 | የአካል ጉዳት አይነት? | ---------------------------------------------- |  |

**ክፍል 2 (A). PHQ-9**

|  | ላለፉት ሁለት ሳምንታት ከነዚህ ከምዘረዝራቸው ችግሮች ውስጥ /በየትኞቹ ተቸግረው/ እንደ ነበር | የለም  (0) | አልፎ አልፎ ብቻ (1) | በዛ ላሉ ጊዜ  (2) | ከሞላ ጎደል በየቀኑ (3) |
| --- | --- | --- | --- | --- | --- |
| ጥ501 | ላለፉት ሁለት ሳምንታት የዕለት ተዕለት ተግባርዎን ለማከናወን /ለመስራት/ ያለዎት ተነሳሽነት ወይም ፍላጎት በጣም ቀንሶ ነበር? |  |  |  |  |
| ጥ502 | ላለፉት ሁለት ሳምንታት የመከፋት የመደበር ወይም ተስፋ የመቁረጥ ስሜት ይሰማዎት ነበር? |  |  |  |  |
| ጥ503 | ላለፉት ሁለት ሳምንታት እንቅልፍ አልወስድ ብልዎት ወይም በደንብ መተኛት አቅትዎት ይቸገሩ ነበር? ወይም እንቅልፍ በዝቶብዎት ይቸገሩ ነበር? |  |  |  |  |
| ጥ504 | ላለፉት ሁለት ሳምንታት የድካም ወይም የአቅም ማነስ ስሜት ይሰማዎት ነበር? |  |  |  |  |
| ጥ505 | ላለፉት ሁለት ሳምንታት የምግብ ፍላጎትዎ ቀንሶ ነበር? ወይም ከተለመደውበላይ ጨምሮ ነበር? |  |  |  |  |
| ጥ506 | ላለፉት ሁለት ሳምንታት ራስዎን የመጥላት ወይም ዋጋ የለኝም የማለት ወይም ራሴንም ሆነ ቤተሰቤን አሳዝኛለሁ የሚል ስሜት ተሰምትዎት ነበር? |  |  |  |  |
| ጥ507 | ላለፉት ሁለት ሳምንታት በሚሰሩት ስራ ላይ ሃሳብዎን ለመሰብሰብ/ትኩረት መስጠት አስቸግርዎት ነበር? /ለምሳሌ ከሰዎች ጋር ሲጨዋወቱ ትኩረት ስጥቶ ማዳመጥ/? |  |  |  |  |
| ጥ508 | ላለፉት ሁለት ሳምንታት ለሌሎች ሰዎች እስከ ሚታወቅ ድረስ በእንቅስቃሴዎ ወይም በንግግርዎ በጣም ቀስብለው ነበር? ወይም ለሌሎች ሰዎች እስከ ሚታወቅ ድረስ መረጋጋት አቅቶዎት አንድ ቦታ አርፎ መቀመጥ ወይመ መቆም እስከ ማይችሉ ሆነው ነበር ? |  |  |  |  |
| ጥ509 | ላለፉት ሁለት ሳምንታት ከምኖር ብሞት ይሻለኛል ብለው አስበው ወይም ራስዎን በሆነ መንገድ ሊጎዱ አስበው ነበር? |  |  |  |  |
| ጥ510 | \| ከተዘረዘሩት ችግሮች ውስጥ አንዳቸውም አዎ የሚሌ መልስ ከተሰጠ የሚከተለውን ይጠይቁ፡፡ በነዚህ ችግሮች ምክንያት ስራዎን መስራት የቤት ኃሊፊነትዎትን መወጣት ወይም ከሰዎች ጋር ተስማምተው መኖር ምን ያህሌ አስቸጋሪ ሆኖብ ዎት ነበር? \| \| --- \| | \| በጭራሽ አልተቸገርኩም \| \| --- \|   (0) | \| በመጠኑ ተቸግሬ ነበር \| \| --- \|   (1) | \| በጣም ተቸግሬ ነበር \| \| --- \|   (2) | \| እጅግ በጣም ተቸግሬ ነበር \| \| --- \|   (3) |

**ክፍል 3**:ከኮቪድ ጋር የተያያዘ ጭንቀት የተመለከተ መጠይቅ

ይህ መጠይቅ የጤና ባለሙያ እርስዎ ምን እንደሚሰማዎት እንዲያውቁ ይረዳቸዋል ፡፡ እርስዎ ምን እንደተሰማዎት በበለጠ በሚገልፀው መልስ ላይ እያንዳንዱን ጥያቄ ያንብቡ እና ‹X› ን ያክብቡ ፡፡ ለእያንዳንዱ ጥያቄ አንድ መልስ ብቻ ያመልክቱ።

| **ተ.ቁ** | **ጥያቄ** | **መልስ** | | | |
| --- | --- | --- | --- | --- | --- |
|  |  | በጭራሽ እርግጠኛ አይደለሁም | ብዙ ቀናት | ከግማሽ ቀን በላይ | በየቀኑ ማለት ይቻላል |
| ጥ601 | የመረበሽ ወይም የጭንቀት ስሜት | 0 | 1 | 2 | 3 |
| ጥ602 | ጭንቀትዎን ማቆም ወይም መቆጣጠር አለመቻል | 0 | 1 | 2 | 3 |
| ጥ603 | ስለ የተለያዩ ነገሮች በጣም መጨነቅ | 0 | 1 | 2 | 3 |
| ጥ604 | ዘና ለማለት ችግር መኖር | 0 | 1 | 2 | 3 |
| ጥ605 | በጣም የውስጥ እረፍት ስለሌለው ለመቀመጥ መቸገር | 0 | 1 | 2 | 3 |
| ጥ606 | በቀላሉ መበሳጨት ወይም የተበሳጨ መሆን | 0 | 1 | 2 | 3 |
| ጥ607 | አንድ አስደንጋጭ ነገር ሊከሰት እንደሚችል የመሰለ ፍርሃት ይሰማኛል | 0 | 1 | 2 | 3 |

**ክፍል 4: ስለ ኮቪድ- 19 እዉቀትን የሚመለከቱ መጠይቆች**

| No | ጥያቄዎች | መልስ |
| --- | --- | --- |
| ጥ701 | ኮቪድ-19 ከአፍና አፍንጫ በሚዎጡ የተለያዩ ብናኞች ይተላለፋል ብለው ያስባሉ? | 1. አዎ 2. የለም 3. አላውቅም |
| ጥ702 | ኮቪድ-19 በትንፋሽ ይተላለፋል ብለው ያስባሉ? | 1.አዎ 2. የለም 3. አላውቅም |
| ጥ703 | በኮቪድ-19 በተበከሉ ቁሳቁሶች ይተላለፋል ብለው ያስባሉ?? | 1.አዎ 2. የለም 3. አላውቅም |
| ጥ704 | በኮቪድ-19 የሚጠቁ ሰዎች ቁጥር ከጊዜ ወደ ጊዜ እየጨመረ መምጣቱን ያውቃሉ? | 1.አዎ 2. አዎ 3. አላውቅም |
| ጥ705 | በኮቪድ-19 የሚሞቱ ሰዎች ቁጥር ከጊዜ ወደ ጊዜ እየጨመረ መምጣቱን ያውቃሉ? | 1. አዎ 2. የለም 3. አላውቅም |

**ክፍል 5: ሱስ የሚያመጡ የንጥረነገር ሁኔታ ጥያቄዎች (አልኮሆል መጠጥ ፣ጫት መቃም፣ ሲጋራ ማጨስ)**

| ተ.ቁ | ጥያቄ | አማራጭ መልሶች | ኮድ |
| --- | --- | --- | --- |
| 201. | በህይወት ዘመነወ ሳብስታንሶቸን(አደንዛዝ እጾቸን) ተጠቅመው ያውቃሉ ? | 1. አዎ 2. አልተጠቀምኩም |  |
| 202 | ለጥያቄ 601 መልስዎ አዎ ከሆነ የትኞቹን ተጠቅመዋል ? | 1. አልኮል 2. ሲጋራ  3. ጫት 4. ሌላ ካለ ይግለጹ------ |  |
| 203 | ባለፉት ሶስት ወራት ሳብስታንሶቸን(አደንዛዝ እጾቸን)ተጠቅመው ያውቃሉ ? | 1. አዎ 2. አልተጠቀምኩም |  |
| 204 | ለጥያቄ 603 መልስዎ አዎ ከሆነ የትኞቹን ተጠቅመዋል? | 1. አልኮል 2. ሲጋራ  3. ጫት 4. ሌላ ካለ ይግለጹ---------- |  |

**ክፍል 6፡- የእንቅልፍ እጦት ችግር ጋር የተያያዘ መጠይቅ**

ሰባት ጥያቄዎች አሉት ፡፡ አጠቃላይ ውጤቱን ለማግኘት ሰባቱ መልሶች ተጨምረዋል። ለእያንዳንዱ ጥያቄ እባክዎን መልስዎን በተሻለ ሁኔታ የሚገልጽ ቁጥር ያክብቡ ፡፡እባክዎን የአሁኑን የእንቅልፍ ችግርዎን ​​(ለምሳሌ ያለፉት 2 ሳምንቶች) ደረጃ ይስጡ ፡፡

|  | የእንቅልፍ ችግር | ምንም | መለስተኛ | መካከለኛ | ከባድ | በጣም ከባድ |
| --- | --- | --- | --- | --- | --- | --- |
| ጥ301 | ለመተኛት መቸገር | 0 | 1 | 2 | 3 | 4 |
| ጥ302 | ተኝቶ አለመቆየት | 0 | 1 | 2 | 3 | 4 |
| ጥ303 | በጣም ቀደም ብሎ ከእንቅልፍ መንቃት | 0 | 1 | 2 | 3 | 4 |
| ጥ304 | አሁን ካለው የእንቅልፍ ሁኔታዎ ጋር ምን ያህል ረክተዋል/አረኩም? | በጣም ረክቻለሁ  0 | ረክቻለሁ    1 | በመጠኑ ረክቻለሁ  2 | አልረካሁም  3 | በጣም አልረካሁም  4 |
| ጥ305 | የእንቅልፍ ችግርዎ ህይወትዎን ከመጉዳት አንፃር ለሌሎች እንዴት ይታወቃል? | በጭራሽ አይታወቅም  0 | በጥቂቱ  1 | በመጠኑም ቢሆን  2 | በጣም  3 | በጣም ብዙ  4 |
| ጥ306 | አሁን ስላለው የእንቅልፍ ችግርዎ ምን ያህል ተጨንቀዋል? | በጭራሽ አልጨነቅም  0 | ትንሽ  1 | በመጠኑም ቢሆን  2 | በጣም  3 | በጣም ተጨንቄአለሁ  4 |
| ጥ307 | አሁን ላይ ያለው የእንቅልፍ ችግር የእለት ተእለት እንቅስቃሴዎን (ለምሳሌ የቀን ድካም ፣ የስሜት ሁኔታ ፣ በዕለት ተዕለት ሥራዎች ውስጥ የመስራት ችሎታ ፣ ትኩረትዎ ፣ የማስወስ ችሎታ ፣ ወዘተ.)ምን ያህል ያውከዋል ብለው ያስባሉ? | በጭራሽ አይታወክም  0 | ትንሽ  1 | በተወሰነ ደረጃ  2 | ብዙ  3 | በጣም ይታወካል  4 |

.

**ክፍል7፡ ችግሮችን የመቋቋም ዘዴ መጠይቆች**

| ተ.ቁ     \|  \| \| --- \| | ጥያቄዎች |  |  |  |  |  |
| --- | --- | --- | --- | --- | --- | --- | --- |
|  |  | መልሶች | | | | |
|  |  | Does not describe me at all | Does not describe me | Neutral | Describes me | Describes me very well |
| ጥ801 | የሚያስጭቅ ነገር ሲገጥመዎት ከጭንቀቱ ለመላቀቅ መፍትሄዎችን ይፈልጋሉ | በጭራሽ | እኔን አይገልጽም | አላዉቅም | እፈልጋለሁ | በጣም እፈልጋለሁ |
| ጥ802 | ምንም ነገር ቢፈጠር መቆጣጠር እችላለሁ ብየ አምናለሁ ብለው ያሰባሉ.. | በጭራሸ | አላስብም | አላዉቅም | አስባለሁ | በጣም አስባለሁ |
| ጥ803 | በችግሩ ምክንያት ለማጣችዉ ነገሮች ሌሎች አማራጮችን እጠቀማለሁ ብለዉ ያሰባሉ. | በጭራሸ | እኔን አይገልጽም | አላዉቅም | አስባለሁ | በጣም አስባለሁ |
| ጥ804 | ቀጣይ የሆኑ ችግሮች ቢገጥሙኝ ችግሮችን በመጋፈጥ የሚቀጥሉ ይመስለዎታል. | በጭራሸ | አይመስለኝም | አላዉቅም | ይመስለኛል | በጣም ይመስለኛል |
